# Insomnia and Endothelial Function – The HUNT 3 Fitness Study

**DOI:** 10.1371/journal.pone.0050933

**Published:** 2012-12-06

**Authors:** Linn B. Strand, Lars E. Laugsand, Eli-Anne Skaug, Øyvind Ellingsen, Erik Madssen, Ulrik Wisløff, Lars Vatten, Imre Janszky

**Affiliations:** 1 Department of Public Health and General Practice, Faculty of Medicine, Norwegian University of Science and Technology, Trondheim, Norway; 2 KG Jebsen Center of Exercise in Medicine, Department of Circulation and Medical Imaging, Norwegian University of Science and Technology, Trondheim, Norway; 3 Department of Cardiology, St. Olavs Hospital, Trondheim, Norway; 4 Department of Public Health Sciences, Karolinska Institutet, Stockholm, Sweden; University of Hong Kong, China

## Abstract

**Background:**

Insomnia is associated with increased risk of coronary heart disease (CHD), but the underlying mechanisms are not understood. To our knowledge, no previous studies have examined insomnia in relation to endothelial function, an indicator of preclinical atherosclerosis. Our aim was to assess the association of insomnia with endothelial function in a large population based study of healthy individuals.

**Methods:**

A total of 4 739 participants free from known cardiovascular or pulmonary diseases, cancer, and sarcoidosis, and who were not using antihypertensive medication were included in the study. They reported how often they had experienced difficulties falling asleep at night, repeated awakenings during the night, early awakenings without being able to go back to sleep, and daytime sleepiness. Endothelial function was measured by flow mediated dilation (FMD) derived from the brachial artery.

**Results:**

We found no consistent association between the insomnia symptoms and endothelial function in multiadjusted models, but individual insomnia symptoms may be related to endothelial function. Among women who reported early awakenings, endothelial function may be lower than in women without this symptom (p = 0.03).

**Conclusions:**

This study provided no evidence that endothelial function, an early indicator of atherosclerosis, is an important linking factor between insomnia and CHD. Further studies are needed to explore the complex interrelation between sleep and cardiovascular pathology.

## Introduction

Insomnia is a subjective feeling of having difficulties initiating or maintaining sleep or having a feeling of non-restorative sleep [1]. Increasing evidence suggests that insomnia is associated with subsequent coronary heart disease (CHD) in populations initially free of heart disease [2]–[4]. Previously, we found a dose-dependent association between number of insomnia symptoms and the risk of acute myocardial infarction which was stronger in women than in men [3]. However, the nature of this association is not yet clear. Atherosclerosis, the underlying pathophysiological mechanism of CHD, is known to develop decades before the first clinical symptoms. It is possible that both insomnia and subsequent CHD are associated with subclinical manifestations of atherosclerosis. Since insomnia is a common and potentially treatable condition, it is important to understand if insomnia is a causal factor or only a correlate of CHD.

The endothelium is a thin layer of cells that forms the inner surface of the blood vessel, forming an interface between circulating blood and the rest of the vessel wall. Healthy endothelial cells control the passage of materials and white blood cells in and out of the blood stream, prevent blood clotting and control blood pressure by releasing nitric oxide (NO) promoting vasodilation [5]. Impaired endothelial function is an indicator of preclinical atherosclerosis [6]–[8] and is characterized by decreased activity of endothelium-derived NO, causing vasoconstriction, platelet aggregation, and thrombus formation, all of which are important factors in the development of atherosclerosis [9]. Endothelial function has also consistently been shown to predict CHD [10]–[14].

**Figure 1:**
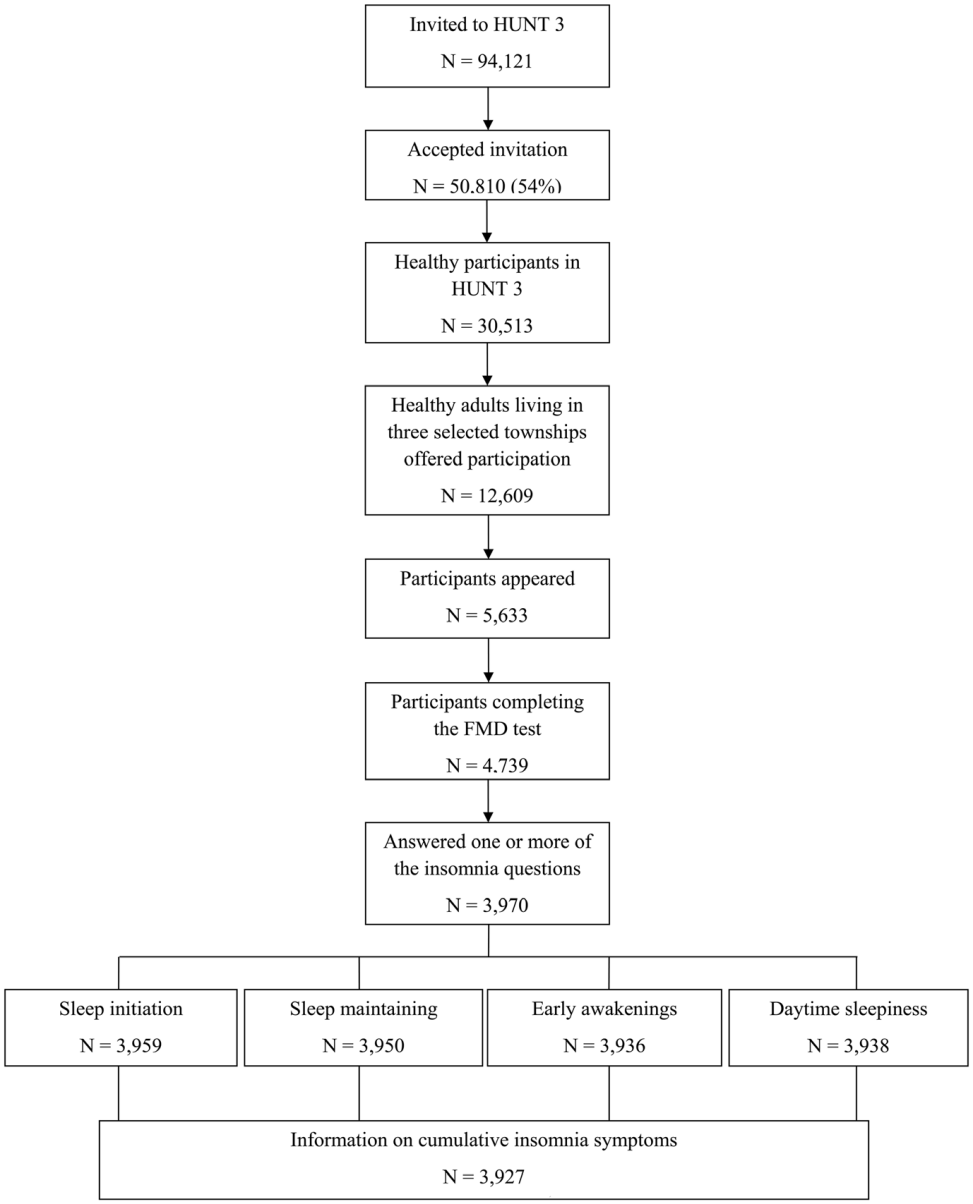
The selection process.

To our knowledge, no previous studies have examined insomnia in relation to endothelial function. If an association was found, it would provide a plausible pathway linking insomnia to CHD. Accordingly, our aim was to assess the association of insomnia with endothelial function when controlled for potentially confounding factors, including age, marital status, education, sleep-disordered breathing, and snoring, established cardiovascular risk factors such as smoking, alcohol consumption, physical activity and BMI, and measures of psychological distress like depression or anxiety.

**Table 1:** Characteristics according to cumulative number of insomnia for women.

		Cumulative insomnia symptoms		
Variable	n	0	1–2	3–4
	N = 2,144	N = 1,468	N = 566	N = 110
Age (years)	2,144	49.1 (13.4)	49.5 (13.6)	51.6 (13.1)
BMI (kg/m^2^)	2,142	25.5 (3.7)	25.5 (4.1)	26.0 (4.4)
HADS anxiety score	2,139	3.5 (2.7)	5.4 (3.4)	6.7 (3.9)
HADS depression score	2,141	2.2 (2.2)	3.2 (2.8)	4.5 (3.3)
Physical activity index	1.986	3.7 (2.5)	3.6 (2.5)	3.3 (3.0)
FMD (%)	2,144	5.3 (4.5)	5.3 (4.4)	5.1 (4.5)
Smoking (%)				
Never	1,052	53.1	43.0	48.8
Previous	581	26.3	31.5	27.4
Current	483	20.6	25.6	23.8
Marital status (%)				
Never married	460	21.0	23.3	18.2
Married	1,316	62.7	59.5	54.6
Separated/divorced/widowed	365	16.3	17.1	27.3
Sleep-disordered breathing (%)				
Never/almost never	2,042	97.5	94.5	96.6
Sometimes	63	2.3	4.6	3.0
Several times a week	8	0.1	0.9	0.4
Snoring (%)				
Never/almost never	1,416	69.6	62.9	61.8
Sometimes	537	25.0	25.9	31.8
Several times a week	147	5.5	11.2	6.7
Alcohol intake (%)				
Abstainers	865	50.7	52.5	48.9
Light drinkers	652	39.7	35.3	38.6
Moderate drinkers	139	7.8	9.5	6.8
Heavy drinkers	37	1.7	2.7	5.7
Education (%)				
Primary	277	11.7	14.0	24.8
Secondary	987	45.3	47.8	49.5
Tertiary	873	43.0	38.2	25.7

## Materials and Methods

### Ethics Statement

All participants gave written informed consent. This research was approved by the Norwegian research ethics committee.

**Table 2:** Characteristics according to cumulative number of insomnia for men.

		Cumulative insomnia symptoms		
Variable	n	0	1–2	3–4
	N = 1,783	n = 1,345	n = 380	n = 58
Age (years)	1,783	50.0 (13.2)	51.4 (13.5)	51.6 (11.3)
BMI (kg/m^2^)	1,782	26.6 (3.0)	26.8 (3.4)	27.1 (3.1)
HADS anxiety score	1,769	2.9 (2.5)	4.8 (3.4)	6.5 (4.1)
HADS depression score	1,769	2.6 (2.5)	4.1 (3.0)	5.7 (3.6)
Physical activity index	1,557	3.7 (2.8)	3.4 (2.6)	3.4 (3.1)
FMD (%)	1,783	4.3 (3.8)	4.0 (3.9)	5.0 (4.1)
Smoking (%)				
Never	901	53.0	45.7	49.1
Previous	488	26.6	32.1	29.1
Current	366	20.4	22.2	21.8
Marital status (%)				
Never married	444	25.2	23.3	31.0
Married	1,127	63.8	63.1	55.2
Separated/divorced/widowed	208	11.1	13.5	13.8
Sleep-disordered breathing (%)				
Never/almost never	1,543	88.9	83.2	80.7
Sometimes	164	8.8	11.0	10.5
Several times a week	58	2.3	5.9	8.8
Snoring (%)				
Never/almost never	735	42.1	38.8	44.8
Sometimes	700	40.9	36.7	25.9
Several times a week	337	17.0	24.5	29.3
Alcohol intake (%)				
Abstainers	285	16.1	18.8	25.5
Light drinkers	710	42.7	41.6	36.4
Moderate drinkers	430	25.7	24.5	30.9
Heavy drinkers	255	15.5	15.1	7.3
Education (%)				
Primary	191	10.2	12.5	12.1
Secondary	989	53.8	50.8	63.8
Tertiary	595	33.0	36.7	24.1

### Data Collection

Between October 2006 and June 2008, the entire adult population of Nord-Trøndelag County in Norway was invited to participate in the third wave of the Nord-Trøndelag Health Study (HUNT-3). Approximately 54% of those who were invited, attended the study (n = 51 000). Information was collected by self-administered questionnaires, a clinical examination and blood samples. Self-reported health status, use of tobacco and alcohol, dietary items, use of medication, and information on sleep, physical activity, and education were included in the questionnaire. Anthropometry, including measurements of height, weight, and waist and hip circumference, was recorded, and blood pressure and serum lipids were measured.

**Table 3:** Least square means and 95% confidence intervals for the insomnia symptoms and flow mediated dilation (%) in women.

			Model 1		Model 2		Model 3	
			Mean	95% CI	Mean	95% CI	Mean	95% CI
**Sleep initiation**								
Never/almost never			5.42	5.13–5.70	5.43	5.10–5.76	5.40	5.07–5.74
Occasionally			5.18	4.91–5.47	5.30	4.96–5.63	5.33	4.99–5.66
Several times/week			5.25	4.69–5.81	5.50	4.81–6.20	5.57	4.86–6.28
*P- value for linear trend*			*0.35*		*0.89*		*0.88*	
*P- value for quadratic trend*			*0.49*		*0.51*		*0.54*	
**Repeated awakenings**								
Never/almost never		5.40		5.07–5.73	5.54	5.16–5.92	5.52	5.13–5.91
Occasionally		5.29		5.03–5.56	5.27	4.95–5.59	5.29	4.97–5.61
Several times/week		5.19		4.76–5.61	5.35	4.83–5.87	5.38	4.86–5.91
*P- value for linear trend*		*0.44*			*0.45*		*0.59*	
*P- value for quadratic trend*		*0.99*			*0.45*		*0.49*	
**Early awakenings**								
Never/almost never	5.47			5.20–5.73	5.58	5.27–5.89	5.57	5.26–5.90
Occasionally	5.17			4.88–5.46	5.28	4.93–5.64	5.30	4.94–5.66
Several times/week	5.12			4.50–5.75	4.56	3.78–5.34	4.57	3.78–5.36
*P- value for linear trend*	*0.15*				*0.02*		*0.03*	
*P- value for quadratic trend*	*0.59*				*0.44*		*0.42*	
**Daytime sleepiness**								
Never/almost never	5.27			4.91–5.62	5.26	4.83–5.69	5.19	4.76–5.63
Occasionally	5.26			5.02–5.50	5.28	5.00–5.57	5.29	5.01–5.58
Several times/week	5.68			5.15–6.22	6.04	5.43–6.65	6.17	5.54–6.80
*P- value for linear trend*	*0.33*				*0.10*		*0.04*	
*P- value for quadratic trend*	*0.28*				*0.12*		*0.10*	

Model 1: Adjusted for age and age squared.

Model 2: Adjusted for age, age squared, marital status, education, smoking, alcohol consumption, physical activity index, BMI, sleep-disordered breathing, snoring.

Model 3: Adjusted for the same variables as in model 2, and depression score and anxiety score.

After excluding 20 533 participants (39%) known to have cardiovascular or pulmonary diseases, cancer, and sarcoidosis, and who were using antihypertensive medication, 30 513 participants were eligible for a separate study called the Fitness study [15], [16]. Among them, 12 609 participants from three selected towns were invited, and a total of 5 633 participants (44.6%) appeared at the test site. After further exclusion of 97 participants (1.7%) with cardiovascular disease or hypertension and 797 participants for other reasons (e.g. pain, illness or reluctance to carry out the test), 4 739 participants completed the endothelial function test. The participants also responded to a physical activity questionnaire. The selection process is illustrated in Figure 1.

**Table 4:** Least square means and 95% confidence intervals for the insomnia symptoms and flow mediated dilation (%) in men.

				Model 1					Model 2		Model 3	
				Mean	95% CI				Mean	95% CI	Mean	95% CI
**Sleep initiation**												
Never/almost never				4.29	4.10–4.52			4.35		4.10–4.61	4.34	4.09–4.60
Occasionally				4.27	3.97–4.57			4.33		3.99–4.66	4.33	3.99–4.67
Several times/week				3.65	2.88–4.41			3.35		2.46–4.25	3.40	2.48–4.31
*P- value for linear trend*				*0.28*				*0.17*			*0.24*	
*P- value for quadratic trend*				*0.33*				*0.11*			*0.12*	
**Repeated awakenings**												
Never/almost never			4.15		3.87–4.42			4.18		3.88–4.48	4.18	3.88–4.48
Occasionally			4.33		4.10–4.60			4.39		4.08–4.69	4.39	4.08–4.69
Several times/week			4.34		3.86–4.81			4.38		3.85–4.91	4.38	3.85–4.91
*P- value for linear trend*			*0.38*					*0.40*			*0.29*	
*P- value for quadratic trend*			*0.66*					*0.63*			*0.65*	
**Early awakenings**												
Never/almost never		4.20			3.96–4.45		4.21			3.94–4.47	4.16	3.89–4.43
Occasionally		4.19			3.90–4.49		4.26			3.93–4.59	4.29	3.96–4.63
Several times/week		4.88			4.02–5.50		4.99			4.31–5.67	5.13	4.43–5.82
*P- value for linear trend*		*0.18*					*0.10*				*0.04*	
*P- value for quadratic trend*		*0.12*					*0.18*				*0.16*	
**Daytime sleepiness**												
Never/almost never	4.45				4.13–4.77	4.48				4.13–4.83	4.45	4.09–4.80
Occasionally	4.16				3.93–4.39	4.17				3.91–4.43	4.17	3.91–4.43
Several times/week	4.14				3.58–4.70	4.40				3.75–5.06	4.48	3.80–5.16
*P- value for linear trend*	*0.18*					*0.41*					*0.58*	
*P- value for quadratic trend*	*0.50*					*0.24*					*0.21*	

Model 1: Adjusted for age and age squared.

Model 2: Adjusted for age, age squared, marital status, education, smoking, alcohol consumption, physical activity index, BMI, sleep-disordered breathing, snoring.

Model 3: Adjusted for the same variables as in model 2, and depression score and anxiety score.

### Insomnia

The participants answered how often during the last three months they had experienced difficulties falling asleep at night, repeated awakenings during the night, and early awakenings without being able to go back to sleep, and daytime sleepiness. The response categories were “never/almost never”, “sometimes” or “several times a week”. These questions differed slightly from the insomnia questions asked in HUNT-2 (1996–1997) which our previous study [3] was based upon. The questionnaire in HUNT-2 included three items related to insomnia and the participants answered how often the last month they had difficulties falling asleep at night, early awakenings without being able to go back to sleep (with the response options never/occasionally, often/almost every night), in addition to how often they suffered from poor sleep, with the response options “never or a few times a year/1–2 times per month/about once a week/more than once a week”.

**Table 5:** Least square means and 95% confidence intervals for the cumulative insomnia symptoms and flow mediated dilation (%).

		Model 1		Model 2		Model 3	
Insomnia symptoms	n	Mean	95% CI	Mean	95% CI	Mean	95% CI
***Women***							
0	1,468	5.31	5.08–5.53	5.33	5.06–5.59	5.32	5.05–5.59
1–2	566	5.29	4.93–5.65	5.57	5.12–6.01	5.61	5.15–6.06
3–4	110	5.28	4.46–6.11	5.16	4.17–6.14	5.26	4.26–6.26
*P- value for linear trend*		*0.92*		*0.72*		*0.55*	
*P- value for quadratic trend*		*0.99*		*0.35*		*0.35*	
***Men***							
0	1,345	4.28	4.08–4.49	4.30	4.07–4.52	4.27	4.04–4.50
1–2	380	4.04	3.65–4.42	4.14	3.69–4.58	4.20	3.75–6.66
3–4	58	5.01	4.03–5.99	5.15	4.04–6.26	5.32	4.18–6.46
*P- value for linear trend*		*0.96*		*0.65*		*0.39*	
*P- value for quadratic trend*		*0.06*		*0.11*		*0.11*	

Model 1: Adjusted for age and age squared.

Model 2: Adjusted for age, age squared, marital status, education, smoking, alcohol consumption, physical activity index, BMI, sleep-disordered breathing, snoring.

Model 3: Adjusted for the same variables as in model 2, and depression score and anxiety score.

In total, 82.9% of the participants in the fitness study (n = 3 927) answered all the insomnia questions. The separate response rates for the questions about difficulties falling asleep at night, repeated awakenings during the night, early awakenings without being able to go back to sleep, and daytime sleepiness, were 83.5% (n = 3 959), 83.4% (n = 3 950), 83.1% (n = 3 936), and 83.1% (n = 3 938), respectively. These response rates largely reflect the overall response rate of the HUNT-3 questionnaire that included the insomnia questions.

Information was also collected on other aspects of sleep: loud snoring, sleep-disordered breathing, sweating while asleep, waking up with a headache and having an uncomfortable feeling in the legs.

### Flow Mediated Dilation (FMD)

All the participants were asked to refrain from food and coffee, smoking and dipping tobacco during the last four hours before testing. The test was performed with the participant lying down, in a dark room with neutral temperature and minimal noise. Measurements were performed by ultrasonography (Vivid-ι, GE healthcare, USA) with 3 point ECG. A 12 Mhz linear array transducer visualized the left brachial artery in the longitudinal plane above the antecubital fossa. Baseline diameter was measured after 10 minutes of supine rest. Arterial occlusion was created by a cuff placed at the forearm [17], [18], inflated at 250 mmHg for 5 minutes, before abruptly released. Blood flow was estimated by pulsed Doppler velocity and recorded ten seconds after cuff deflation. Post diameter was measured 60 sec after cuff deflation. All arterial diameters were recorded at the peak of the R-wave in ECG, to avoid confounding of cyclic changes in the arterial dimension. The mean of three measurements (intima to intima) was recorded using optical callipers with 0.1-mm resolution. Shear rate was calculated as blood velocity (cm/s) divided by vessel diameter (cm). The difference in baseline diameter and post diameter was used as maximal dilation of the artery; yielding FMD expressed as per cent change.

### Physical Activity

The participants answered a physical activity questionnaire that included questions about frequency, intensity and duration. The frequency question was stated as “How often do you exercise?” with the response options “Never”, “Less than once a week”, “Once a week”, “Two to three times a week” or “Almost every day”. The question related to intensity was stated as “How hard do you exercise?” with the response options “No sweat or heavy breathing”, “Sweat and heavy breathing”, or “Push myself to exhaustion”. Duration of the exercise was stated as “How long does each session last?” with the response options “Less than 15 minutes”, “Between 15 and 29 minutes”, “Between 30 and 60 minutes”, or “More than 60 minutes”. Frequency, intensity and duration were combined to form a physical activity index. We recoded the frequency scale to approximate number of times per week (i.e. “0”, “0.5”, “1”, “2.5”, or “5”), the intensity scale to “1”, “2”, or “3”, and the duration scale to the approximate number of hours per session (i.e. “0.10”, “0.38”, “0.75”, and “1.00”). The physical activity index was the product of the recoded frequency, intensity and duration scales. This method of calculating physical activity level has been reported and validated previously [19].

### Clinical Information

Clinical information on weight, height, and blood pressure was collected by trained nurses. Systolic and diastolic blood pressure was measured three times (Dinamap 845XT Criticon) and the average of the second and third measurement was used in the analysis. Height was measured to the nearest 1 cm and weight to the nearest 0.5 kg. The participants wore light clothes and no shoes during these measurements. Body mass index (BMI) was calculated as weight (in kilograms) divided by height squared (in meters).

Socio-demographic (i.e. sex, age, and marital status) and lifestyle factors (i.e. smoking and alcohol intake) were collected by questionnaires. Marital status was categorised into never married, married, or separated/divorced/widowed, and education was categorised into whether they had completed primary and lower secondary school, upper secondary school, or university. The participants were defined as either never smokers, previous smokers or current smokers. In relation to alcohol consumption, the participants reported how many glasses of beer, wine and spirits they usually consumed over a two-week period. From this information, we categorised the participants into abstainers, light drinkers (0–1 drinks per day), moderate drinkers (above 1 but below 2 drinks per day), or heavy drinkers (2 or more drinks per day).

### Anxiety and Depression

To assess symptoms of anxiety and depression, the Hospital Anxiety and Depression Scale (HADS) was used. The questionnaire consists of 14 questions (7 for anxiety and 7 for depression) with a four point scale ranging from 0 (not at all) to 3 (very often). Responses are summed to provide separate scores for symptoms of anxiety and depression with possible scores ranging from 0 to 21 for each scale. Higher scores indicate greater likelihood of depression or anxiety [20]. The Hospital Anxiety and Depression Scale is a useful tool in the assessment of symptom severity both in hospital settings and in primary health care [21]. The psychometric properties of the scale have been validated previously in the HUNT study [22].

### Participant Characteristics

Characteristics of the participants according to cumulative number of insomnia symptoms are presented in Table 1 and Table 2. For both women and men, participants with insomnia symptoms tended to be older and heavier than participants without insomnia symptoms. They also had lower physical activity level and were less educated. In both sexes, depression and anxiety scores increased with increasing number of insomnia symptoms. Compared to women without insomnia symptoms, women with insomnia symptoms were more likely to be heavy drinkers and separated, divorced or widowed. For men, but not in women, the prevalence of insomnia symptoms increased with increasing severity of sleep-disordered breathing and snoring.

### Statistical Analysis

The data were analysed using general linear models. We assessed each insomnia symptom using the original response categories. First, we adjusted for both age and square of age (Model 1). In the next model, we further adjusted for marital status, education, smoking, alcohol consumption, BMI, physical activity index, sleep-disordered breathing, and snoring (Model 2). Lastly, we included depression score, and anxiety score in addition to factors included in Model 2 (Model 3). We calculated least square means of FMD with corresponding 95% confidence intervals for each category of insomnia symptoms. Participants with no insomnia complaints were the reference group. In analyses of trend, we assigned a value from 1–3 to the insomnia variables representing “never/almost never”, “sometimes”, and “several times a week”, respectively. We treated these variables as continuous variables to test for linear trend. In separate analyses, we included a quadratic term to assess non-linear trends.

The insomnia symptoms were also dichotomized so we could assess the association of cumulative number of symptoms with FMD. Participants with the most frequent symptoms (i.e. those experiencing the symptom several times a week) were defined as having the respective symptom. To test the cumulative association of insomnia symptoms, we calculated the least square mean associated with increasing number of dichotomized insomnia symptoms (i.e. 0, 1–2, and 3–4). In this analysis, we excluded participants with missing information on one or more of the insomnia variables.

As a sensitivity analysis we repeated the original analysis after excluding participants who reported sleep-disordered breathing.

To assess possible effect modification we conducted stratified analyses as well as performed formal tests of interactions with sex, age (i.e. below and above 50 years), and BMI (i.e. below and above 30 kg/m^2^).

In these analyses, we found indication for a possible sex difference in the association between some of the insomnia symptoms and FMD. The p-values for an interaction by gender in relation to sleep initiation, frequent awakenings, early awakenings and daytime sleepiness were 0.565, 0.172, 0.002 and 0.036, respectively. Because of these findings, and due to the well-known sex differences in endothelial function and in the prevalence of insomnia [16], [23], [24] all models were run separately for women and men.

All statistical analyses were conducted using STATA 12 for Windows (Stata corp., College Station Texas).

## Results

We found no evidence for an association of having difficulties initiating sleep or maintaining sleep with FMD in either women (Table 3) or men (Table 4).

Women experiencing early awakenings several times/week had an estimated FMD of 4.56% (95% CI 3.78–5.34) compared to 5.58% (95% CI 5.27–5.89) in women without the symptom after adjusting for age, marital status, education, smoking, alcohol consumption, self-reported physical activity, body mass index (BMI), sleep-disordered breathing, and snoring (p for trend = 0.02). Further adjustment for anxiety and depression did not change the estimates.

Among men, there was no apparent association between daytime sleepiness and estimated FMD in any of the models. For women however, we found that those who reported a feeling of daytime sleepiness several times/week had an estimated FMD of 6.17% (95% CI 5.54–6.80) compared to 5.19% (95% CI 4.76–5.63) in other women (fully adjusted model, p for trend = 0.04).

For both women and men, we found no consistent associations between the cumulative number of insomnia symptoms and FMD in any of the models (Table 5). We obtained similar results after excluding participants who reported sleep-disordered breathing sometimes and several times/week (n = 293, results not shown).

We found no evidence of effect modification of age or BMI for any of the symptoms in any of the models (results not shown).

## Discussion

To the best of our knowledge, this is the first study of insomnia and endothelial function. Overall, we found no consistent associations between insomnia and endothelial function. Thus, we could not provide clear evidence for the early indicator of atherosclerosis acting as a link between insomnia and CHD.

A few small-scale experimental studies have examined the possible vascular effects of acute sleep deprivation [25], [26]. In those studies, the investigators observed a decrease in vascular reactivity after short term sleep deprivation.

Some previous studies have examined the association of more chronic sleep deprivation with endothelial function [27], [28]. One study [28] reported that mean endothelial function had decreased from 7.4% to 3.7% before and after a 4-week period with chronic sleep deprivation. Another study [27] found an inverse relation between mean sleep duration and forearm blood flow responses to infusion of an endothelin receptor antagonist, indicating that habitual short sleep duration is associated with impaired endothelial function. However, both studies were small and did not provide sex-specific results. Also, there was no available information on anxiety and depression, two factors that are potentially important confounders in relation to insomnia and cardiovascular diseases, since anxiety and depression are both related to insomnia [29], [30] and to the risk of cardiac events [31].

In our previous analyses of the HUNT-2 study, we found that experiencing insomnia symptoms increased the risk of incident AMI [3]. Recently, endothelial function was collected in HUNT-3, and we were able to explore whether endothelial dysfunction could be a mechanism behind the previously observed association between insomnia and AMI. However, the HUNT-3 study does not have an adequate follow time yet to investigate insomnia and AMI risk in this population.

Our analyses of individual insomnia symptoms suggest that some of the symptoms might be related to endothelial function, and that this relation might differ by sex. Among women we observed an inverse association of early awakenings with endothelial function, but there was an opposite trend among men. Also, women that reported daytime sleepiness had a higher FMD than other women. Of note, a large study reported a reduction in all-cause mortality associated with increasing frequency of insomnia [32]. Therefore, some aspects of insomnia might actually have a protective effect in certain subgroups. Nevertheless, the positive association between daytime sleepiness and endothelial function that we found among women was unexpected and warrants further evaluation in future studies.

The nature of the association of insomnia and CHD is unclear, and it is unknown whether insomnia is a cause or only a marker of increased risk. Endothelial dysfunction, an indicator of atherosclerosis in its earlier, preclinical stage, had no overall consistent association with insomnia in our study. However, insomnia might have an impact on CHD through other pathways, including influence on plaque stability via increasing the level of pro-inflammatory cytokines or on thrombosis via increasing the level of pro-coagulatory factors [33]–[38]. The latter two mechanisms, in contrast to endothelial dysfunction refer to a late stage of atherosclerosis. The increased CHD risk apparent in persons suffering from insomnia [2]–[4], [39], regardless of its exact nature, suggests that insomniacs and their doctors should be aware of increased cardiovascular risk and should be able to recognize symptoms and early manifestations of CHD in an effort to forestall its serious consequences.

### Limitations

Despite of its clear strengths that include the large sample size, the population based design and the use of directly measured FMD, our study also has some important limitations.

It was based on self-reported measures of sleep, and objective measures (i.e. polysomnography) were not available. Insomnia is defined as a subjective feeling of having difficulties falling asleep, remaining asleep or receiving restorative sleep [1], [24] and thus, such problems are not routinely evaluated by polysomnography [40]. However, our evaluation largely reflects the DSM-IV criteria of insomnia [1].

We did not evaluate chronic insomnia symptoms. If an association with endothelial function only result from prolonged exposure to insomnia symptoms, not to only recent exposure, and subjects in our study belonged mainly to the latter group, the association between insomnia and vascular dysfunction might be diluted and difficult to detect.

Information about sleep duration was not available in our study. However, insomnia is a different condition than short sleep duration [32] and people with insomnia symptoms could have normal or long duration of sleep [41]. Also, some people with short sleep duration may not have any insomnia symptoms because there is a large variation between individuals in the duration of sleep that is needed for physiological and psychological restoration [42], [43]. It is also important to recognize that insomnia provides additional information on the quality of sleep. From one study it was reported that the increased risk of CHD associated with short duration of sleep was greatest among those with a sleep disturbance [44]. Thus, only accounting for sleep duration will miss information about subjective quality of sleep that may be important in the assessment of its harmful effects.

In our study, we restricted to individuals free of cardiovascular diseases (CVD) and hypertension. This restriction, as a conditioning on common consequences of cardiovascular risk factors, might introduce a collider stratification bias, which is a frequent problem in similar cross sectional studies [45], [46].

This study may also be subject to self-selection bias. Only 54% of the eligible population responded to the HUNT-3 study and only 44.6% of those invited to the Fitness study chose to participate in the FMD testing. Thus, it might be suspected that only the healthiest accepted the invitation. However, by comparing the participants in the fitness study with a healthy sample of the total HUNT population (i.e. free from cardiovascular or pulmonary diseases, cancer, or sarcoidosis), it was confirmed that the fitness participants were not considerably different from other healthy participants in the HUNT Study with regard to self-reported and measured health variables [15].

We cannot exclude the possibility that the reported insomnia symptoms are caused by underlying pathology and that the insomnia symptoms are secondary to that condition. Because participants in the HUNT-3 study with known cardiovascular or pulmonary diseases, cancer, sarcoidosis, and users of antihypertensive medication were ineligible for our study however, this possibility has been minimised.

It is possible that some of the covariates included in models 2 and 3 may act as mediators in the association of insomnia with endothelial function. For example alcohol consumption near bed time may be used as sleep aid. However, adjustment for these factors had very limited impact on the regression coefficients.

Our study was performed on an apparently healthy, socio-economically homogenous population and the results cannot be directly generalized to less healthy populations or to countries on different latitudes, different socioeconomic status or with different sleeping habits.

### Conclusions

In this large population based study of apparently healthy individuals, we found no consistent association of insomnia with FMD. Thus, endothelial function did not provide a clear link between insomnia and subsequent CHD. As it is an early indicator of preclinical atherosclerosis and a significant determinant of cardiovascular diseases, our findings may be important in the complex interrelation between sleep and the cardiovascular system. The sex-specific differences related to some of the insomnia symptoms warrant further investigation.
